# Stage of breast cancer at diagnosis in New Zealand: impacts of socio-demographic factors, breast cancer screening and biology

**DOI:** 10.1186/s12885-016-2177-5

**Published:** 2016-02-19

**Authors:** Sanjeewa Seneviratne, Ross Lawrenson, Vernon Harvey, Reena Ramsaroop, Mark Elwood, Nina Scott, Diana Sarfati, Ian Campbell

**Affiliations:** Waikato Clinical School, University of Auckland, Hamilton, New Zealand; Department of Surgery, University of Colombo, Colombo, Sri Lanka; Auckland District Health Board, Auckland, New Zealand; School of Population Health, University of Auckland, Auckland, New Zealand; Māori Health Services, Waikato District Health Board, Hamilton, New Zealand; University of Otago, Wellington, New Zealand

**Keywords:** Breast cancer, Stage, Ethnicity, Inequity

## Abstract

**Background:**

Examination of factors associated with late stage diagnosis of breast cancer is useful to identify areas which are amenable to intervention. This study analyses trends in cancer stage at diagnosis and impact of socio-demographic, cancer biological and screening characteristics on cancer stage in a population-based series of women with invasive breast cancer in New Zealand.

**Methods:**

All women diagnosed with invasive breast cancer between 2000 and 2013 were identified from two regional breast cancer registries. Factors associated with advanced (stages III and IV) and metastatic (stage IV) cancer at diagnosis were analysed in univariate and multivariate models adjusting for covariates.

**Results:**

Of the 12390 women included in this study 2448 (19.7 %) were advanced and 575 (4.6 %) were metastatic at diagnosis. Māori (OR = 1.86, 1.39-2.49) and Pacific (OR = 2.81, 2.03-3.87) compared with NZ European ethnicity, other urban (OR = 2.00, 1.37-2.92) compared with main urban residency and non-screen (OR = 6.03, 4.41-8.24) compared with screen detection were significantly associated with metastatic cancer at diagnosis in multivariate analysis. A steady increase in the rate of metastatic cancer was seen which has increased from 3.8 % during 2000-2003 to 5.0 % during 2010-2013 period (*p* = 0.042).

**Conclusions:**

Providing equitable high quality primary care and increasing mammographic screening coverage needs to be looked at as possible avenues to reduce late-stage cancer at diagnosis and to reduce ethnic, socioeconomic and geographical disparities in stage of breast cancer at diagnosis in New Zealand.

## Background

Breast cancer is the commonest cause of cancer in New Zealand women (excluding non-melanoma skin cancer) and accounts for approximately 3000 diagnoses and 600 deaths per year [1]. One of the most important factors in predicting survival from breast cancer is the stage at diagnosis. Women with early stage disease have an excellent prognosis while those with metastatic disease at diagnosis have a 5-year survival of around 20 % [2]. Stage at diagnosis can be influenced by the diagnostic pathway and the characteristics of the tumour [3]. The diagnostic pathway is important – population based screening with mammography has been shown to increase the proportion of women diagnosed with early breast cancer [4].

In New Zealand, late diagnosis with advanced or metastatic disease has been associated with Māori ethnicity and social deprivation [5]. Differences by ethnicity and social deprivation have important associations in other countries [6–9], while in some countries it has been shown that women living outside main urban areas are more likely to be diagnosed with advanced disease [10]. The reason why women from certain demographic groups may present late may be due to factors related to the women e.g. health literacy, psychosocial factors, etc., [11] or system issues causing diagnostic delays such as a shortage of primary care physicians [12] and difficulty accessing diagnostic facilities [13]. Furthermore, community-level determinants which include health policy, health care delivery system, and community risk factors have also been observed to be contributing to socioeconomic and geographic variations in breast cancer stage at diagnosis [14]. Another cause of advanced stage at diagnosis may be the biology of the cancer – some types of cancer are more aggressive and are more likely to metastasize early [15].

It is important when looking at a population of women with breast cancer to examine the associations with late diagnosis to identify which factors may be amenable to intervention. This study assesses the importance of socio-demographic factors, breast cancer screening and biological factors in explaining differences in cancer stage at diagnosis in a population-based series of female patients diagnosed with invasive breast cancer in New Zealand.

## Methods

### Data sources

Data were obtained from the Auckland (ABCR) and Waikato Breast Cancer Registries (WBCR), which are prospective population based databases that capture almost 100 % of the newly diagnosed breast cancers in the respective regions since 2000. These two registries cover an area that includes over 40 % of the total New Zealand population. In general, this population resembles average New Zealand population in terms of ethnicity, socioeconomic status and urban/rural residential distribution. Completeness and accuracy of the data included in these registries have been validated previously [16, 17]. Data from the two registries were linked with the New Zealand Cancer Registry (NZCR) and the National Mortality Collection (NMC).

### Study population

All women with primary breast cancer diagnosed over a 13-year period between 01/06/2000 and 31/05/2013 were identified from the two registries. This included a total of 14469 breast cancers [12390 (85.6 %) invasive and 2079 (14.4 %) in situ cancers]. Of this 12390 women with invasive, primary breast cancer were included in the analyses.

### Healthcare system in New Zealand

New Zealand has a publicly funded national health system that provides specialist and hospital care to all citizens without patient charges. Parallel to the public system, there are a variety of private hospital facilities available, which are mostly funded through insurance schemes. The primary health care system in New Zealand is highly subsidized, but patient co-payment is also substantial. For instance, a visit to a general practitioner on average may cost between NZ$20 and NZ$50 for an adult. A national breast cancer screening programme, BreastScreen Aotearoa provides free biannual breast cancer screening for all women aged 45–69 years, and has operated since 1999.

### Study covariates

Patient ethnicity was identified from the breast cancer registries or where it was not available from these registries it was obtained from the NZCR or the NMC, as per the Ministry of Health ethnicity data protocols [18]. Ethnicity was categorized into NZ European, Māori, Pacific, Asian and Other. Socioeconomic deprivation was classified according to the New Zealand Deprivation Index 2006 (NZDep2006) [19]. The NZDep2006 assigns small residential areas a deprivation decile on a scale of 1 to 10 based on nine socio-economic variables measured during the 2006 population census; decile1-least deprived, decile10-most deprived. Urban/rural residential status of each woman was categorized into main urban, other urban (independent or satellite urban) and rural based on the New Zealand Statistics urban/rural classification system [20]. These variables were selected on the basis of theoretical relevance and empirical evidence of their utility in assessing the impact of socio-demographic factors on a variety of health outcomes including cancer [21]. Cancer stage at diagnosis was defined according to the Tumour, Node, and Metastasis (TNM) system [22] and was categorized into early (TNM stage groups I and II), advanced (stage groups III and IV) and metastatic (stage group IV) for analysis. Invasive tumour grade was defined according to the Elston and Ellis modified Scarff-Bloom-Richardson breast cancer grading system [23]. Oestrogen (ER) and progesterone (PR) receptor status was determined based on the results of immunohistochemistry tests and classified as positive or negative. HER-2 status was based on Fluorescent In-Situ Hybridization (FISH) test or when this was not available, on immunohistochemistry [24].

### Statistical analysis

Univariate differences in distribution of factors among groups of interest were tested by using Chi squared (*χ*²) test for trend or by linear-by-linear association test [25]. Unconditional logistic regression models were used to estimate the contribution of covariates towards advanced or metastatic cancer at diagnosis in multivariate analyses. All statistical analyses were performed in SPSS (Version 22) (18).

### Ethics approval

Both ABCR and WBCR function with ethics approval from the New Zealand Northern ‘A’ Health and Disability Ethics Committee. This required individual patient consent from patients for their data to be included. Since 2012, the consent process was waived off by the same ethics committee as it was noted that for data from these registries to be more useful at a national level all patients with breast cancer are needed to be included. Additionally ethical approval for this study was obtained from the same New Zealand Northern ‘A’ Health and Disability Ethics Committee (Ref. No. 12/NTA/42).

## Results

Of the 12390 women included in this study 9630 (77.7 %) were from the Auckland Region and 2755 (22.3 %) from the Waikato. The mean age of the population was 57 years. 8972 (73.3 %) were NZ European, 1162 (9.5 %) were Māori, 809 (6.6 %) were Pacific, 984 (8 %) Asian and 311 (2.5 %) belonged to Other ethnic groups. Ethnicity data were unavailable for 152 (1.2 %) women. Māori, Pacific and Asian women were significantly younger than the NZ European women, in keeping with the younger age structure of Māori, Pacific and Asian populations in New Zealand [26]. Staging data were missing for 5 (0.04 %) while tumour grade was missing for 5.5 % of study women. Information on hormone receptor status was not available for 2.4 % of the study population. HER-2 status was not available for 23.2 % of the women, a majority (84.2 %) of whom were diagnosed with breast cancer prior to 2006.

Distribution of socio-demographic and tumour characteristics by stage at diagnosis are summarized in Table 1. Proportion of metastatic disease have increased from 3.8 % to 5.0 % over the study period while the rate of stage I cancers has increased from 42.2 % to 45.6 %. A corresponding reduction was seen for rates of stage II and III cancers (from 38.0 % to 35.9 % and 15.7 % to 13.6 %, respectively). Age younger than 40 and older than 70 years were significantly associated with advanced and metastatic breast cancer at diagnosis compared with women aged between 40 to 69 years, a majority of whom are within the breast cancer screening age. NZ European and Asian women tended to be diagnosed at an earlier stage, compared with Māori and Pacific women. Māori and Pacific women were around two and three times more likely respectively, to be diagnosed with metastatic disease compared with NZ European women (3.9 % vs. 7.6 % and 10.9 %, respectively). Over a third (33.7 %) of the cancers in Pacific women and a quarter of the cancers in Māori (26.1 %) were advanced (stage III or IV) at diagnosis compared with less than a fifth in NZ European (18.3 %) and Asian (17.7 %) women.

**Table 1:** Distribution of selected characteristics by percentage among 12,390 female breast cancer patients diagnosed in New Zealand during 2000–2013

Characteristic	Stage I		Stage II		Stage III		Stage IV			Total	
	*n*	%	*n*	%	*n*	%	*n*	%	*p*	*n*	%
	5362	43.3 %	4575	36.9 %	1873	15.1 %	575	4.6 %		12385	100.0 %
Age											
<40	204	25.4 %	333	41.5 %	218	27.1 %	48	6.0 %	<0.001	803	6.5 %
40–49	1053	39.2 %	1029	38.3 %	492	18.3 %	114	4.2 %		2688	21.7 %
50–59	1603	47.8 %	1144	34.1 %	485	14.5 %	122	3.6 %		3354	27.1 %
60–69	1579	55.2 %	866	30.3 %	311	10.9 %	105	3.7 %		2861	23.1 %
70–79	606	38.7 %	652	41.7 %	202	12.9 %	104	6.6 %		1564	12.6 %
80+	317	28.4 %	551	49.4 %	165	14.8 %	82	7.4 %		1115	9.0 %
Ethnicity											
NZ European	4024	44.9 %	3302	36.8 %	1291	14.4 %	351	3.9 %	<0.001	8968	73.3 %
Māori	430	37.0 %	428	36.8 %	216	18.6 %	88	7.6 %		1162	9.5 %
Pacific	217	26.8 %	319	39.4 %	186	23.0 %	87	10.8 %		809	6.6 %
Asian	439	44.7 %	370	37.6 %	140	14.2 %	34	3.5 %		983	8.0 %
Other	161	51.8 %	105	33.8 %	31	10.0 %	14	4.5 %		311	2.5 %
Unknown	91		51		9		1			152	(1.2 %)
Menopausal status											
Pre	1291	36.1 %	1402	39.2 %	738	20.6 %	150	4.2 %	<0.001	3581	29.9 %
Peri	288	45.2 %	239	37.5 %	93	14.6 %	17	2.7 %		637	5.3 %
Post	3554	45.8 %	2821	36.4 %	998	12.9 %	385	5.0 %		7758	64.8 %
Unknown	221		111		44		23			399	(3.2 %)
Year of diagnosis											
2000–2003	1262	42.4 %	1131	38.0 %	467	15.7 %	113	3.8 %	0.001	2973	24.0 %
2004–2006	1097	41.1 %	1016	38.0 %	436	16.3 %	122	4.6 %		2671	21.6 %
2007–2009	1309	43.3 %	1094	36.2 %	466	15.4 %	156	5.2 %		3025	24.4 %
2010–2013	1694	45.6 %	1334	35.9 %	504	13.6 %	184	5.0 %		3716	30.0 %
Region											
Auckland	4270	44.3 %	3509	36.4 %	1439	14.9 %	412	4.3 %	<0.001	9630	77.8 %
Waikato	1092	39.6 %	1066	38.7 %	434	15.8 %	163	5.9 %		2755	22.2 %
Deprivation											
1–2	1210	46.4 %	957	36.7 %	353	13.5 %	87	3.3 %	<0.001	2607	21.2 %
3–4	963	47.4 %	726	35.8 %	268	13.2 %	73	3.6 %		2030	16.5 %
5–6	1166	44.4 %	985	37.5 %	372	14.2 %	105	4.0 %		2628	21.4 %
7–8	1020	40.8 %	913	36.5 %	429	17.2 %	139	5.6 %		2501	20.4 %
9–10	940	37.5 %	958	38.3 %	440	17.6 %	166	6.6 %		2504	20.4 %
Unknown	63		36		11		5			115	(0.9 %)
Urban rural											
Main urban	4064	43.7 %	3421	36.8 %	1417	15.2 %	391	4.2 %	<0.001	9293	75.8 %
Other urban	328	45.6 %	262	36.4 %	86	12.0 %	43	6.0 %		719	5.9 %
Rural	901	40.0 %	855	38.0 %	358	15.9 %	136	6.0 %		2250	18.3 %
Unknown	69		37		12		5			123	(1.0 %)
Mode of detection											
Screen	3196	67.2 %	1211	25.5 %	296	6.2 %	51	1.1 %	<0.001	4754	38.4 %
Non-screen	2166	28.4 %	3364	44.1 %	1577	20.7 %	524	6.9 %		7631	61.6 %
Grade											
I	2003	70.1 %	694	24.3 %	134	4.7 %	26	0.9 %	<0.001	2857	24.4 %
II	2324	42.7 %	2137	39.2 %	821	15.1 %	167	3.1 %		5449	46.5 %
III	858	25.2 %	1548	45.4 %	820	24.1 %	180	5.3 %		3406	29.1 %
Unknown	177		196		98		202			673	(5.4 %)
ER/PR											
ER/PR Positive	4526	46.1 %	3545	36.1 %	1381	14.1 %	373	3.8 %	<0.001	9825	81.3 %
ER & PR Negative	716	31.7 %	928	41.0 %	471	20.8 %	146	6.5 %		2261	18.7 %
Unknown	120		102		21		56			299	(2.4 %)
HER-2											
Positive	465	29.2 %	576	36.2 %	418	26.3 %	132	8.3 %	<0.001	1591	16.7 %
Equivocal	99	48.5 %	73	35.8 %	27	13.2 %	5	2.5 %		204	2.1 %
Negative	3449	44.7 %	2861	37.0 %	1110	14.4 %	302	3.9 %		7722	81.1 %
Unknown	1349		1065		318		136			2868	(23.2 %)
Histology											
Ductal	4378	44.2 %	3644	36.8 %	1476	14.9 %	409	4.1 %	<0.001	9907	81.3 %
Lobular	480	34.3 %	570	40.7 %	281	20.1 %	68	4.9 %		1399	11.5 %
Mixed	19	26.8 %	30	42.3 %	21	29.6 %	1	1.4 %		71	0.6 %
Other	450	55.9 %	255	31.7 %	62	7.7 %	38	4.7 %		805	6.6 %
Unknown	35		76		33		59			203	(1.6 %)

Significantly higher proportions of more advanced cancer, including metastatic cancer were observed in women from high deprivation compared with low deprivation groups and rural compared with urban residing women (Table 1). Proportions of advanced and metastatic cancer were observed to be higher in the Waikato (21.7 % and 5.9 %, respectively) compared with Auckland (19.2 % and 4.3 %, respectively). Further, a greater increase in the proportion of metastatic cancer was observed in the Waikato region (58 %) compared with Auckland (17 %) over the study period. Significantly higher proportions of advanced cancer were seen in non-screen compared with screen detected women and in women receiving treatment from public compared with non-public hospitals. Greater proportions of cancers with adverse prognostic characteristics including higher grade, oestrogen (ER) and progesterone receptor (PR) negativity and human epidermal growth factor type-2 (HER-2) positivity were advanced or metastatic at diagnosis compared with lower grade, ER/PR positive and HER-2 negative cancers, respectively.

Multivariate logistic regression models were used to assess the importance of study variables in explaining advanced and metastatic cancer at diagnosis and are shown in Table 2. Patients for whom information on tumour stage (*n* = 5) were not available were excluded from regression analyses. Advanced cancer at diagnosis was significantly associated with Māori [Odds ratio (OR = 1.27, 1.08–1.49)] and Pacific (OR = 1.72, 1.43–2.06) compared with NZ European ethnicity, higher socioeconomic deprivation (*p* < 0.001) and non-screen compared with screen detection (OR = 3.79, 3.33–4.34).

**Table 2:** Multivariate analysis for factors associated with advanced and metastatic breast cancer versus early breast cancer at diagnosis, 2000–2013

Characteristic	Univariate						Adjusted					
	OR	Advanced 95% CI	p	OR	Metastatic 95% CI	p	OR	Advanced 95% CI	p	OR	Metastatic 95% CI	p
Age												
<40	1.70	1.43–2.02		1.63	1.15–2.31		1.10	0.92–1.32		1.03	0.70–1.50	
40–49	Ref		0.000	Ref		0.000	Ref		0.000	Ref		0.021
50–59	0.76	0.67–0.86		0.81	0.62–1.05		1.10	0.96–1.26		1.24	0.93–1.65	
60–69	0.59	0.51–0.67		0.78	0.60–1.03		0.90	0.78–1.05		1.19	0.88–1.61	
70–79	0.84	0.72–0.98		1.51	1.15–1.99		0.85	0.72–1.00		1.50	1.10–2.04	
80+	0.98	0.83–1.16		1.72	1.29–2.32		0.76	0.63–0.91		0.84	0.59–1.21	
Year of diagnosis												
2000–2003	Ref		0.068	Ref		0.079	Ref		0.311	Ref		0.042
2004–2006	1.09	0.96–1.24		1.22	0.94–1.59		0.93	0.80–1.08		1.19	0.87–1.64	
2007–2009	1.07	0.94–1.21		1.37	1.07–1.76		0.95	0.81–1.12		1.51	1.10–2.07	
2010–2013	0.94	0.83–1.06		1.29	1.01–1.65		0.87	0.75–1.02		1.44	1.06–1.96	
Region												
Auckland	Ref		0.004	Ref		0.000	Ref		0.741	Ref		0.903
Waikato	1.16	1.05–1.29		1.43	1.18–1.72		1.03	0.85–1.24		0.98	0.68–1.42	
Ethnicity												
NZ European	Ref			Ref			Ref			Ref		
Maori	1.58	1.37–1.82	0.000	2.14	1.68–2.73	0.000	1.27	1.08–1.49	0.004	1.86	1.39–2.49	0.000
Pacific	2.27	1.95–2.65	0.000	3.38	2.64–4.35	0.000	1.72	1.43–2.06	0.000	2.81	2.03–3.87	0.000
Asian	0.96	0.81–1.14	0.639	0.88	0.61–1.26	0.475	0.81	0.67–0.98	0.026	0.90	0.61–1.33	0.609
Other	0.76	0.55–1.04	0.755	1.10	0.64–1.90	0.737	0.87	0.62–1.23	0.426	1.56	0.86–2.83	0.146
Unknown	0.31	0.17–0.60	0.000	0.15	0.02–1.05	0.056	0.37	0.19–0.72	0.003	0.22	0.03–1.63	0.139
Deprivation												
1–2	Ref		0.000	Ref		0.000	Ref		0.000	Ref		0.017
3–4	0.99	0.85–1.16		1.08	0.78–1.48		0.96	0.81–1.14		0.84	0.59–1.19	
5–6	1.09	0.95–1.26		1.21	0.91–1.63		1.05	0.90–1.23		0.99	0.71–1.36	
7–8	1.45	1.26–1.66		1.79	1.36–2.36		1.31	1.12–1.53		1.40	1.02–1.92	
9–10	1.57	1.37–1.80		2.18	1.67–2.84		1.28	1.09–1.51		1.40	1.01–1.92	
Unknown	0.80	0.46–1.36		1.26	0.50–3.18		0.79	0.14–4.47		0.94	0.01–109	
Urban rural												
Main urban	Ref		0.009	Ref		0.001	Ref		0.981	Ref		0.004
Other urban	0.91	0.74–1.10		1.40	1.01–1.93		1.03	0.83–1.29		2.00	1.37–2.92	
Rural	1.16	1.04–1.30		1.48	1.21–1.81		1.01	0.82–1.25		1.22	0.83–1.82	
Unknown	0.66	0.40–1.11		0.90	0.37–2.28		0.78	0.15–4.18		0.40	0.01–46.1	
Mode of detection												
Screen	Ref		0.000	Ref		0.000	Ref		0.000	Ref		0.000
Non−screen	4.83	4.28–5.44		8.18	6.13–10.9		3.79	3.33–4.34		6.03	4.41–8.24	
ER/PR status												
ER/PR Positive	Ref		0.000	Ref		0.000	Ref		0.070	Ref		0.620
ER & PR Negative	1.73	1.55–1.92		1.92	1.58–2.34		1.13	0.99–1.28		1.06	0.83–1.36	
Unknown	1.60	1.23–2.08		5.45	4.00–7.45		1.50	1.06–2.14		1.12	0.71–1.79	
Grade												
Grade I	Ref		0.000	Ref		0.000	Ref		0.000	Ref		0.000
Grade II	3.73	3.13–4.44		3.88	2.56–5.88		2.85	2.38–3.40		2.72	1.78–4.14	
Grade III	7.00	5.88–8.35		7.76	5.12–11.7		4.13	3.30–5.00		3.85	2.47–5.99	
Unknown	13.6	10.8–16.9		56.2	36.8–85.7		12.7	9.82–16.4		49.6	31.2–79.1	
HER-2 status												
Negative	Ref		0.000	Ref		0.000	Ref		0.000	Ref		0.000
Equivocal	0.83	0.57–1.22		0.61	0.25–1.49		0.73	0.48–1.10		0.46	0.18–1.19	
Positive	2.36	2.10–2.66		2.65	2.13–3.28		1.57	1.38–1.79		1.80	1.41–2.31	
Unknown	0.84	0.75–0.94		1.18	0.96–1.45		0.63	0.54–0.74		0.63	0.45–0.87	

Odds ratios for metastatic compared with early stage (i.e., stages I and II) cancer were significantly elevated for Māori (OR = 1.86, 1.39–2.49) and Pacific (OR = 2.81, 2.03–3.87) women, but not for Asian (OR = 0.90, 0.61–1.33) or Other (OR = 1.56, 0.86–2.83) women, relative to NZ European women (Table 2). Non-screen compared with screen detection (OR = 6.03, 4.41–8.24), other urban compared with main urban residency (OR = 2.00, 1.37–2.92), higher socioeconomic deprivation and later year of diagnosis were also significantly associated with metastatic cancer at diagnosis.

Associations between stage at diagnosis and, ethnicity and sociodemographic factors were additionally analysed by year category to identify trends over time (data not shown). However, no significant differences in these associations were observed by year category. As socioeconomic and geographic variables included missing data, regression analysis was repeated using only cases with complete data for all variables. The results were almost identical to the full dataset regression model, and are not presented in this report. Imputation of missing values was not undertaken due to the similarity of these results.

## Discussion

This study has shown major and significant differences in stage of breast cancer at diagnosis by ethnicity, socioeconomic status and by urban/rural residency in New Zealand. We also observed a significant rise in the proportion of metastatic breast cancer although the overall proportion of advanced breast cancer did not show a significant change over the 13 year study period.

Differences in rate of advanced or metastatic breast cancer by ethnicity and socioeconomic status may be due, in part, to delays in responding to breast symptoms which may differ between ethnic and socioeconomic groups. Such differences occur because of differences in access to care or due to patient factors which include health literacy, health seeking behaviours or psycho-social factors [27–29]. Structural organization of the healthcare system also contributes to these disparities by forming barriers to access primary health care services for women of minority ethnicity, low socioeconomic status or non-urban residency [30]. For example, a short supply of primary care physicians has been associated with late stage of breast cancer at diagnosis [12]. Difficulties in accessing primary care due lack of general practitioners (GP) especially outside main urban areas, the costs associated with accessing GPs and barriers due to services not being culturally safe are well-established in New Zealand [31, 32]. A relative lack of GPs commonly seen in rural and low socioeconomic areas may lead to patient overload and hence, a lower quality of care. For instance, if doctors only have a short time to see patients then they may be less likely to undertake routine examinations or ask about the presence of breast lumps. All these factors contribute to a delay and more advanced cancer stage at diagnosis which leads to poor cancer outcomes [33].

Improved delivery of primary health care and increasing mammographic breast cancer screening coverage, as observed in New Zealand over the last two decades [31], would be expected to have reduced the rate of advanced and metastatic cancer at diagnosis. In contrast, the present study observed a steady increase in the proportion of metastatic breast cancer at diagnosis. A similar scenario is reported from several other developed countries including the USA, where the rate of metastatic cancer has remained static or has steadily increased over the last two to three decades [34]. This rise has been more pronounced among women younger than 50 years [35]. It is unclear whether the observed increase in metastatic cancer in our study population is an actual increase or an apparent increase due to several other reasons. For instance, stage migration due to improvements in diagnostic imaging technology, or increasing use of imaging studies for staging over time might have placed patients in a higher stage group at diagnosis [36]. Stage migration tends to occur from an adjacent category (e.g., from stage I or stage III to stage II) and usually from a lower to the next higher stage category (e.g., stage III to stage IV). In keeping with this, our study also observed a reduction in the proportion of stage III cancer which was approximately equal to the increase in rate of metastatic cancer. Regardless, the trajectory of the proportion of metastatic breast cancer predicts a likely increase in the number of women being diagnosed with metastatic breast cancer in the future.

Some researchers argue that the lack of a decrease in rate of metastatic breast cancer observed in many developed countries is a reflection of the ineffectiveness of programmes aimed at improving early diagnosis of breast cancer which include mammographic breast cancer screening [34]. However, it is possible that a majority of these women who are diagnosed with metastatic breast cancer are the very same women who have poor access to health care services, and hence fail to be captured by mammographic screening programmes. As seen in the present study, only a very small proportion of cancers among women who are diagnosed through mammographic screening are metastatic at diagnosis and the vast majority of metastatic cancers are diagnosed in women with symptomatic cancer. Furthermore, many researchers have estimated that approximately a half of the reduction in breast cancer mortality observed in the last two decades in the developed world has been due to widespread use of mammographic screening [37, 38]. Therefore it is unlikely that mammographic screening is ineffective, rather it is the lack of penetration of these programmes into populations of deprived women who are likely to gain a greater benefit that limits its effectiveness at population level.

Increasing rate of metastatic cancer poses several challenges. First, the healthcare system needs to be geared up to deal with women with metastatic cancer whose numbers are likely to increase. Many of these women nowadays have prolonged survivals due to improved treatments [39] which will further increase the disease burden on the health system. In addition to controlling metastatic disease with various treatments, these women will also require expanded services to provide long-term palliative care and psycho-social support. Second, it is essential to identify the reasons for the failure of present health strategies to reduce the rates of advanced and metastatic breast cancer in New Zealand. Identification of sub-groups of women who are at risk of being diagnosed with metastatic cancer, as shown in this study may help develop strategies specifically targeting these groups to promote early diagnosis.

Potential limitations of this study include use of area level deprivation as a proxy measure for individual socioeconomic status and missing data for some of the included variables. The NZDep2006 has been validated as an accurate proxy measure for assessment of socioeconomic deprivation for epidemiological studies [40], although it inherently has a limited precision to predict individual deprivation. Further, we did not include data on health insurance status or lifestyle factors (e.g., weight or body mass index, physical activity, diet, etc.,) or BRCA or other genetic panel-type testing that can influence breast cancer risk and possibly likelihood of screening in our analyses as these were not available from the registries. The analysis performed including only complete dataset yielded very similar results to the results shown; hence missing data are unlikely to have affected the reported findings significantly. Strengths of this study include the completeness of the sample, which essentially included 100 % of newly diagnosed cancers over the study period, and the comprehensive nature of the prospectively collected data from the registries. Hence, these study findings are likely to be representative of breast cancer in New Zealand.

In conclusion, this study has shown significant differences in the proportions of advanced and metastatic breast cancer at diagnosis by ethnicity, socioeconomic status and geography. Further, a small but a significant increase in the proportion of metastatic breast cancer was observed over time. While ensuring increasing provisions to manage metastatic breast cancer, steps needed to be instituted to promote early diagnosis to reduce the rate of metastatic breast cancer at diagnosis. Increasing breast cancer screening coverage and increasing health literacy especially among deprived populations who are at a greater risk of being diagnosed with advanced breast cancer may help reduce the rate of metastatic breast cancer at diagnosis and reduce disparities in stage at diagnosis.
